# A Novel Approach for Quantifying the Pharmacological Activity of T-Cell Engagers Utilizing *In Vitro* Time Course Experiments and Streamlined Data Analysis

**DOI:** 10.1208/s12248-021-00637-2

**Published:** 2021-12-03

**Authors:** Arthur Van De Vyver, Miro Eigenmann, Meric Ovacik, Christian Pohl, Sylvia Herter, Tina Weinzierl, Tanja Fauti, Christian Klein, Thorsten Lehr, Marina Bacac, Antje-Christine Walz

**Affiliations:** 1 Roche Pharma Research & Early Development, Pharmaceutical Sciences, Roche Innovation Center Basel, Grenzacherstrasse 124, CH-4070 Basel, Switzerland; 2 Department of Clinical Pharmacy, Saarland University, Saarbrücken, Germany; 3 Preclinical Translational Pharmacokinetics, South San Francisco, CA Genentech, USA; 4 Roche Pharma Research and Early Development, Roche Innovation Center Zürich, Wagistrasse 10, 8952 Schlieren, Switzerland

**Keywords:** CD3-bispecifics, *in vitro* dose–response, MABEL, PKPD, quantitative pharmacology

## Abstract

CD3-bispecific antibodies are a new class of immunotherapeutic drugs against cancer. The pharmacological activity of CD3-bispecifics is typically assessed through *in vitro* assays of cancer cell lines co-cultured with human peripheral blood mononuclear cells (PBMCs). Assay results depend on experimental conditions such as incubation time and the effector-to-target cell ratio, which can hinder robust quantification of pharmacological activity. In order to overcome these limitations, we developed a new, holistic approach for quantification of the *in vitro* dose–response relationship. Our experimental design integrates a time-independent analysis of the dose–response across different time points as an alternative to the static, “snap-shot” analysis based on a single time point commonly used in dose–response assays. We show that the potency values derived from static *in vitro* experiments depend on the incubation time, which leads to inconsistent results across multiple assays and compounds. We compared the potency values from the time-independent analysis with a model-based approach. We find comparably accurate potency estimates from the model-based and time-independent analyses and that the time-independent analysis provides a robust quantification of pharmacological activity. This approach may allow for an improved head-to-head comparison of different compounds and test systems and may prove useful for supporting first-in-human dose selection.

**Supplementary Information:**

The online version contains supplementary material available at 10.1208/s12248-021-00637-2.

## INTRODUCTION

CD3-bispecific antibodies are a growing class of promising therapies in the field of immuno-oncology (1). Since the clinical success with blinatumomab—a CD19 × CD3-bispecific antibody that was approved by the FDA in 2014 for acute lymphoblastic leukemia (2, 3)—a variety of different CD3-bispecific antibodies or antibody fragments have been designed. More than 200 CD3-bispecifics are currently in development as novel cancer immunotherapies (1, 4). CD3-bispecifics activate an anti-cancer immune response by redirecting T-cells to the tumor (5), with promising anti-cancer activity (6–8) in both hematological (9) and solid (10) tumors.

The pharmacological response is based on tumor antigen recognition combined with CD3-mediated T-cell recruitment. This involves a cascade of events including T-cell activation and proliferation, cytokine release with involvement of innate immune cells, and T-cell-mediated tumor lysis (11–13)—distinct biological processes that occur on different time scales (14–16). CD3-bispecifics’ efficacy (tumor cell toxicity) and safety (*e.g.*, cytokine release) are both related to the mechanism of action. These effects can be investigated *in vitro* as a basis for determining the minimum anticipated biological effect level (MABEL) dose for first-in-human (FIH) clinical trials (17). Due to the lack of cross-reactive animal species for some of the CD3-bispecifics (18–20), *in vitro* test systems are often utilized for pharmacological profiling of this class of molecules (20). In addition, *in vitro* analysis is suited to differentiate and to select compounds and is—in conjunction with in vivo PK/PD analysis—an important pillar for human PK/PD prediction (21).

The appropriate pharmacological quantification *in vitro* may help to improve the therapeutic index of these immune agonists, allowing for the selection of the most favorable compounds during early drug discovery. Appropriate quantification is also critical for predicting a clinically relevant, safe, and pharmacologically active starting dose that will reduce the number of patients exposed to subtherapeutic dose levels in FIH studies. This is a key challenge for CD3-bispecific therapy development (17). Based on a retrospective assessment of 17 CD3-bispecifics, Saber and colleagues conclude that there is no generalizable approach for MABEL-based dose selection applicable to all CD3-bispecifics (17). They highlight that FIH dose selection based on 30% *in vitro* pharmacological activity of the most sensitive readout results in doses that are substantially lower than the optimal biological dose, the recommended human dose, or the maximum tolerated dose in patients. In addition, a 2018 FDA workshop on the preclinical and translational safety assessment of CD3-bispecifics concluded that it is still unclear which *in vitro* experimental conditions (*e.g.*, effector-to-target ratio, target cell choice, assay duration, assay endpoints) are most appropriate for quantitative clinical translation and FIH starting dose prediction (22).

In the presented study, we suggest a modified experimental design and data analysis to explore and leverage *in vitro* data and to derive a more robust pharmacological quantification and a more appropriate integration of the multiple drug-induced PD responses that occur on different time scales. Often, the *in vitro* quantification of CD3-bispecifics is done at a single time point. The derived potency value is highly dependent on the incubation time and susceptible to time point selection bias. Consequently, the apparent potency for a specific compound varies from time point to time point (23). We demonstrated that the dose–response analysis derived from static *in vitro* experiments, as traditionally applied, depends on the incubation time and that this “snap-shot” analysis leads to inconsistent results. To overcome this limitation, we developed a more holistic approach for quantification of the *in vitro* dose–response relationship that considers the time course of the pharmacodynamic (PD) responses and that enables *in vitro* comparison of different readouts (*e.g.*, cytokine release and cytotoxicity) or of different test systems (*e.g.*, cancer cell line, organoids from a tumor or healthy tissue). This approach includes an *in vitro* experimental design that allows us to monitor the time course of the pharmacological responses and a subsequent time-independent dose–response analysis integrating all measured time points (24). As a result, we obtain a more robust readout that provides more consistent insights into the pharmacological activity of CD3-bispecifics. We also developed an automated data analysis workflow that can be applied to these large datasets. Finally, we illustrate how the proposed approach can be implemented to compare the pharmacological activity across test systems and compounds and how these pharmacological insights can be utilized in early drug discovery and development.

## MATERIALS AND METHODS

### Materials

The CD3-bispecifics (also called T-cell bispecifics; TCBs) CEA-TCB (cibisatamab), CEACAM5-TCB, and the FolR1-TCB affinity variants were produced in-house (Roche, Basel, Switzerland). Compound characteristics are summarized in Table I. The full list of all materials, cell lines, and reagents can be found in supplemental section S1.1.

**Table I:** Summary of Tested CD3-Bispecifics

Compound	Molecular weight (KDa)	Avidity to TA (nM)	Affinity to CD3 (nM)
CEA-TCB	194	48.6^a^	3.7^b^
CEACAM5-TCB	194	13.12^a^	3.7^b^
FolR1^high^-TCB	194	2.2^b^	3.7^b^
FolR1^low^-TCB	194	60^b^	3.7^b^

^*a*^Determined with FRET as described in Van De Vyver et al*.* (25). ^*b*^Determined with surface plasmon resonance; *TCB*, T-cell bispecific; *kDa*, kilodalton; *TA*, tumor antigen

### Methods

#### Generation of Stably Transduced Cell Lines Expressing Human FolR1

HEK 293 T cells were transduced with lentiviral particles to express human folate receptor 1 (FolR1) on their cell membrane. A high (FolR1^high^) and a low (FolR1^low^) target-expressing variant was created, with a surface density of 505,000 and 20,000 molecules/cell, respectively. Details on the generation of the stably transduced cell lines are summarized in supplemental section S1.2. Cell surface density of FolR1 was determined by QIFIKIT (Agilent Dako).

#### Experimental Design of T-Cell-Dependent Cellular Cytotoxicity Assay

The *in vitro* pharmacology of cibisatamab and CEACAM5-TCB was tested with a T-cell-dependent cellular cytotoxicity (TDCC) assay and flow cytometric analysis for tumor lysis and immune-phenotyping. Additionally, *in vitro* pharmacology of FolR1-TCB variants was tested with an alternative TDCC method that included real-time monitoring of fluorescent tumor cells by incuCyte.

#### FACS Based Assay to Monitor Tumor Cell Count After Treatment with Cibisatamab and CEACAM5-TCB

The experimental protocol for *in vitro* activity testing of cibisatamab has been previously described (25). This protocol was also applied to the *in vitro* testing of CEACAM5-TCB. Briefly, two cell lines expressing the oncofetal antigen CEA (carcinoembryonic antigen), MKN45 (high copy number: 230,000–690,000 CEA per cell) and CX1 (low copy number: 2000–11,000 CEA per cell), were seeded at a density of 30,000 cells/well in an effector-to-target ratio of 10:1 with human PBMCs and incubated at varying concentrations of cibisatamab (0, 6, 32, 160, 800, 4000, 20,000, & 100,000 pM) or CEACAM5-TCB (0, 1, 6, 32, 160, 800, 4000, & 20,000 pM). At 24 h, 48 h, 72 h, 96 h, and 168 h, supernatants were collected for cytokine analysis, and cell pellets were used for flow cytometric analysis. FACS analysis was performed for tumor cell counting and immune-phenotyping of T-cells on the expression of CD3, CD4, CD8, CD25, PD-1, and TIM-3.

#### IncuCyte Assay to Monitor Tumor Cell Cytotoxicity with FolR1-TCB

Staining of tumor cells and PBMCs compatible with incuCyte imaging was performed as per the manufacturer’s instructions (Sartorius). IncuCyte NucLight Rapid Red was used to fluorescently label the nucleus of HEK cells.

A total of 10,000 FolR1-expressing FolR1^high^ or FolR1^low^ tumor cells were seeded in flat-bottom 96-well plates and co-cultured with 100,000 PBMCs in assay medium (RPMI1640 + 20% FCS + 1% GlutaMax). Dilutions of either a high-affinity or a low-affinity FolR1-TCB were added to reach the final drug concentrations (0.5, 5, 50, 500, 5000, and 50,000 pM). For the negative control, 50 μL of assay medium was added. All conditions were performed in triplicate. The co-cultures were incubated over 4 days in a Sartorius incuCyte Zoom (humidified, 37 °C, 5% CO_2_) for automated imaging at 3-h intervals. All co-cultures were duplicated fivefold to allow for supernatant sampling at 18 h, 44 h, 68 h, and 94 h.

#### Cytokine Measurements

At the indicated time points (18 h, 44 h, 68 h, and 94 h), the plates were centrifuged and 25 μL of supernatant was collected from each well. Cytokines IL2, IL6, IL10, IFNγ, and TNFα were measured with a multiplexed cytometric bead array.

##### Statistics and Data Analysis

Experiments were performed in triplicate and data were processed as median values. Where applicable, the experimental data are reported as mean values with corresponding standard error (SEM). Values that are below their lower limit of quantification (LLOQ) are reported as ½ LLOQ. Estimated dose–response parameters are reported with their respective relative standard error (%RSE).

#### Dose–Response Analysis

Dose–response curves were generated based either on a single time point or on the time-independent PD effect using WinNonlin (Phoenix 8.2, Certara). This time-independent response is computed as an area under the curve of effect (AUCE) in the readout for the PD response over time for each tested TCB concentration. For estimation of the potency parameter EC_50_, a simple or inhibitory sigmoidal model was fitted to the data (Eq. (1)).

1 $$E= {E}_{0}+\frac{{E}_{max}*{TCB}^{\gamma }}{{EC}_{50}^{\gamma }+{TCB}^{\gamma }}$$

*E* is the value of the experimental readout. In static analysis, *E* is the actual readout, and in time-independent analysis, *E* is either the calculated *AUCE* value of the readout or the maximum value (*R*_max_) of the readout. *TCB* is the independent variable, corresponding to the concentration of the CD3-bispecific. *E*_*0*_ is the baseline level of the readout or *AUCE*, *E*_*max*_ is the maximum change in *E*, *γ* is the Hill coefficient, and *EC*_*50*_ is the drug concentration resulting in a half-maximum effect. An additive residual error was used to fit Eq. (1) to the respective experimental data. Based on Eq. (1), the concentration leading to 30% pharmacological activity (PA_30%_) was derived as follows:

2 $${PA}_{30\%}= {EC}_{50}*{\left(\frac{30\%}{100\%-30\%}\right)}^{\frac{1}{\gamma }}$$

If a sigmoidal dose–response relationship could not be established, a threshold concentration was estimated by fitting the data to the hockey-stick model (26) (Eq. (3)).

3 $$E= {E}_{0}+S*\left(TCB-{TCB}_{threshold}\right)*{P}^{+}$$

*TCB*_*threshold*_ is the threshold drug concentration for eliciting the PD effect above baseline (*AUCE*_*0*_) and *S* is the change in effect when the drug concentration is greater than the threshold value. *P*^+^ is a derived variable that is zero when drug concentration is below the threshold concentration and one when the drug concentration is above the threshold concentration.

##### Automated Dose–Response Analysis with Python

An automated dose–response analysis was conducted using Python. The data file was curated using *pandas* library. The Python code is provided in a GitHub repository (https://github.com/PKPD-coder/time-independent_analysis_in_vitro.git) and can be updated for other datasets. The workflow of the automated analysis is illustrated in Figure S1. Further details are summarized in supplemental section S2.

#### Predicting Tumor Growth Inhibition with PK/PD Modeling

The observed cell counts from cibisatamab and CEACAM5-TCB were fitted to a delayed tumor kill model (27) in Monolix (version 2019R2, Lixoft, France) to estimate the respective EC_50_ values of tumor cytotoxicity. The tumor growth model (Eq. (8)) assumes a logistic tumor growth with *k*_*g*_ representing the tumor growth rate and K the carrying capacity, which can be interpreted as the maximum tumor cell number that can be reached. The drug effect (*k*_*el*_) is based on a sigmoidal dose–response relationship (Eq. (4)) with *E*_*max*_ representing maximum cytotoxicity and *TCB* representing the actual drug concentration. A delayed drug effect is assumed and described by means of three transit compartments (Eqs. (5)–(6)) with **τ** describing the transit kinetics (27). The model parameters (fixed effects) were estimated and the standard errors of the random effects were fixed to 10% during model fitting. A combined error model was used.

4 $${k}_{el}={E}_{max}*\frac{TCB}{{EC}_{50}+TCB}$$

5 $${dk}_{1}/ dt= \frac{1}{{\boldsymbol{\uptau}}}*({k}_{el}-{k}_{1})$$

6 $${dk}_{2}/ dt= \frac{1}{{\boldsymbol{\uptau}}}*({k}_{1}-{k}_{2})$$

7 $${dk}_{3}/ dt= \frac{1}{{\boldsymbol{\uptau}}}*({k}_{2}-{k}_{3})$$

$$k_1\;(0)=k_2\;(0)=k_3\;(0)=0$$

8 $$\begin{array}{c}dTumor/ dt={k}_{g}*\left(1-\frac{Tumor}{K}\right)-{k}_{3}*Tumor\end{array}$$

#### Trimeric Complex Prediction

The concentration of trimeric complexes formed between the tumor target, CD3 and CD3-bispecific antibody, was estimated under quasi-equilibrium assumptions and based on the equations derived by Schropp and colleagues (28). The corresponding equations are summarized in supplemental section S3 (Eq. S1-S7). A Python script is provided in order to perform the calculation (https://github.com/PKPD-coder/time-independent_analysis_in_vitro.git).

## RESULTS

### *In Vitro* Dose–Response Analysis

**Fig. 1:**
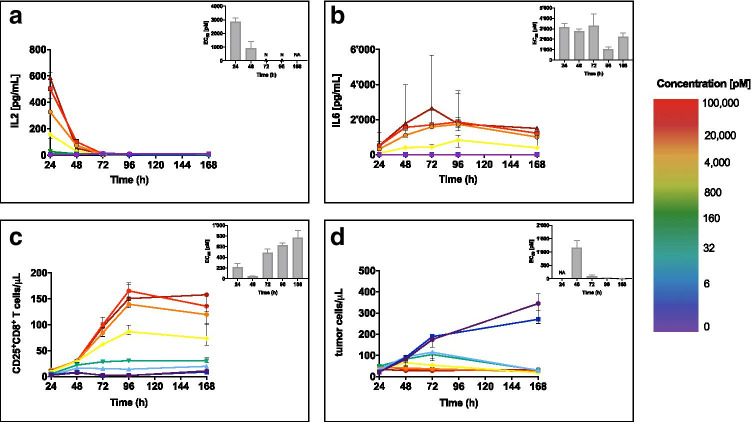
Time course of pharmacodynamic response of cibisatamab tested in a T-cell-dependent cytotoxicity assay on MKN45 tumor cells co-cultured with human PBMC (E:T 10:1). Dose–response over time is shown for **a** IL2 release; **b** IL6 release; **c** T-cell activation measured as CD25 + CD8 + T-cells; and **d** drug-related tumor cell cytotoxicity. The static EC_50_ potency estimates for each time point are displayed in the inset plots. Results are shown as the median and range of the replicates

Using a TDCC assay, we assessed the PD effects of cibisatamab (CEA-TCB) on MKN45 tumor cell lines expressing CEA, co-cultured with human PBMCs *in vitro*. The *in vitro* kinetics of tumor cell cytotoxicity, cytokine release, and T-cell activation induced by cibisatamab are shown in Fig. 1 and Figure S2. Cytokine release is shown for IL2 (Fig. 1a) and IL6 (Fig. 1b), as well as IL10, IFNγ, and TNFα (Figure S2). T-cell activation was monitored by measuring CD8^+^CD25^+^ T-cells (Fig. 1c). Tumor cell killing was captured by monitoring the tumor cell count dynamics in the absence and presence of the drug (Fig. 1d).

For all tested PD readouts, we observed a clear dose–response. PD effects increased with increased drug concentration. Each readout displayed a maximum response at a different time point. Table II summarizes the observed maximum response time (*t*_*max*_) for each readout. IL2 had the fastest response, with a maximum release at the first observed time point (24 h) at all drug concentrations followed by a rapid decline in the presence of constant drug exposure. However, T-cell activation increased over time, reaching a maximum level of activation in CD8^+^CD25^+^ T-cells at 96 h, when IL2 levels were no longer detectable. We observed measurable IL6 released at each time point, with peaks occurring between 48 and 96 h depending on the drug concentration, followed by a slow decrease in IL6 concentration. IL10 (Figure S2H) and IFNγ (Figure S2J) showed a similar release pattern to IL6, while TNFα (Figure S2I) showed a similar release pattern to IL2, with a peak after 24 h followed by a rapid decrease.

**Table II:** Dose–Response Analysis of Cibisatamab Tested on MKN45, Time of Maximal Response, and Dynamic Potency

Parameter	*T*_*max*_ (h)	EC50 (pM) (%RSE)	Hill coefficient (%RSE)
Activated cytotoxic T-cells (CD25^+^CD8^+^)	96	596 (13)	0.91 (11)
Tumor cell cytotoxicity	96	15.7 (27)	2.07 (31)
IL2	24	2280 (16)	1.19 (17)
IL6	72	1501 (16)	1.28 (17)
IL10	48	1890 (17)	1.09 (16)
TNFα	24	1437 (19)	0.88 (15)
IFNγ	48	409 (3.0)	1.49 (3.0)
CD4^+^CD25^+^	96	686 (5.0)	1.24 (5.0)
CD4^+^PD1^+^	168	545 (9.0)	1.22 (10)

*T*_*max*_, time of maximal response; *%RSE*, relative standard error in percentage

The results of the static dose–response analysis are displayed in the insets of Figs. 1a–d showing time-dependent EC_50_ values observed for various PD readouts. Notably, the EC_50_ estimates for each readout varied between 3- and 110-fold across the different time points.

### Time-Independent Quantification of Drug Response

**Fig. 2:**
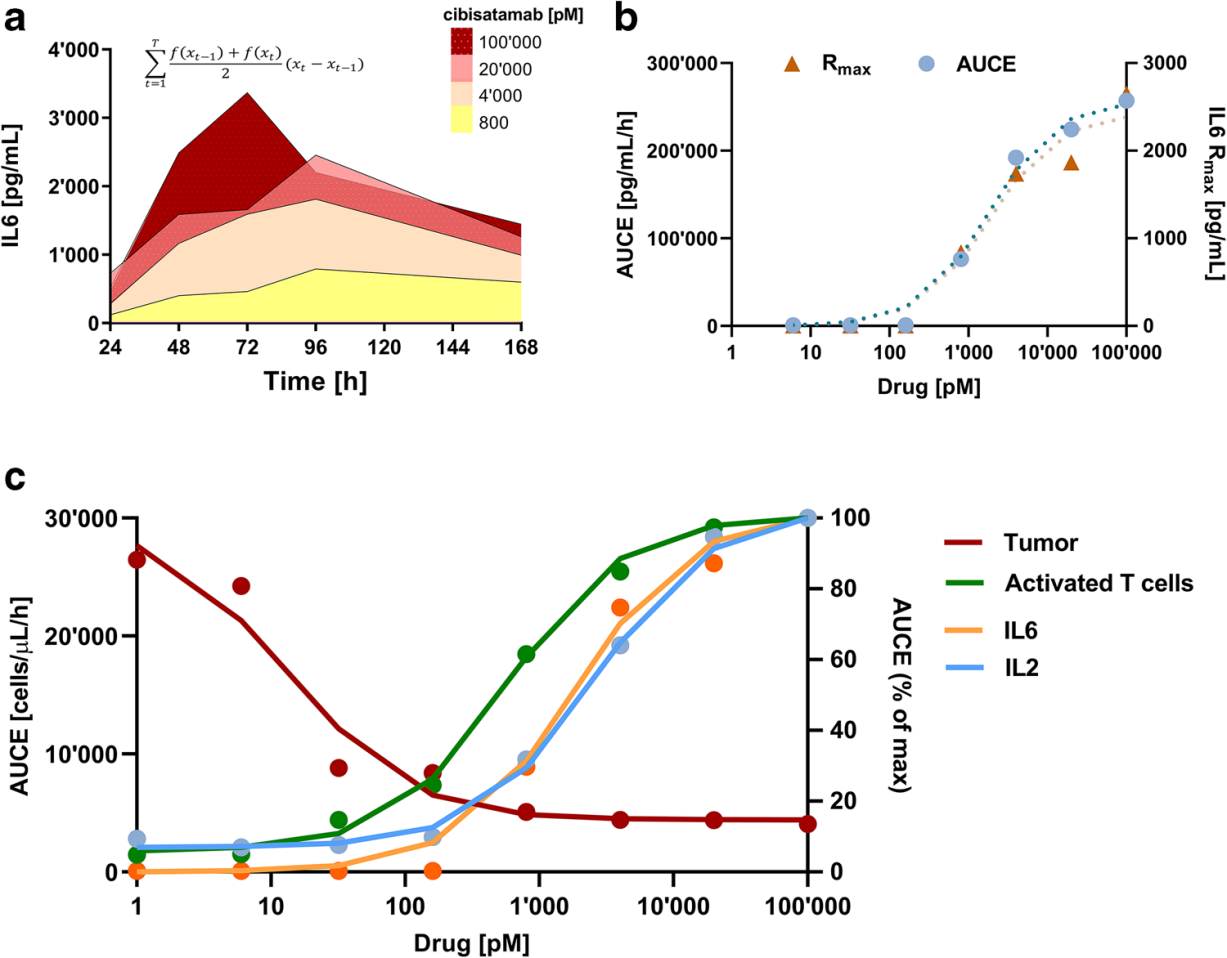
Time-independent analysis workflow example for cibisatamab. **a** Integration with trapezoidal interpolation (inset equation) of dose–response curves for IL6 release over time yields individual area-under-the-effect-curve (AUCE; shaded areas) for each TCB concentration. **b** Overlay of dose–response curves for IL6 release based on AUCE (blue circles) or maximum response (*R*_*max*_, orange triangle). **c** Time-independent dose–response comparison between tumor cell cytotoxicity (red), T-cell activation (green), IL2 (blue), and IL6 (orange) release

Subsequently, we performed a time-independent, two-step *in vitro* analysis to quantify and compare the dose–response on the various PD readouts of the TDCC assays (Fig. 2). We illustrate this approach for IL6. First, we compute the time-independent PD effect as the area-under-the-effect-curve (AUCE) with the trapezoidal rule (Fig. 2a and inset equation). We then fit a sigmoidal drug effect model to the drug concentration-AUCE curve (Fig. 2b). We overlaid this curve with the dose–response of IL6 release (*R*_*max*_), indicating that there is good agreement between estimated exposure–response relationships derived based on AUCE and *R*_*max*_ for IL6. Table II summarizes the estimated EC_50_ values and corresponding Hill coefficients. Figure 2c shows the overlay of the derived pharmacological profiles of tumor cytotoxicity, T-cell activation, and IL2 and IL6 release. Furthermore, for all tested readouts, the dose–response relationship was similar when the time-independent or the maximum effect was used (supplemental Figure S3).

In the tested time frame, tumor cytotoxicity (Fig. 2c, red curve) was the most sensitive readout for cibisatamab, with an EC_50_ 38-fold lower than T-cell activation and approximately 145- and 96-fold lower than IL2 and IL6 release, respectively. In the tested *in vitro* system, maximum tumor cytotoxicity (IC_99_) was reached at a concentration of 155 pM cibisatamab, which approximately corresponds to the EC_5_ for IL2 and IL6 release.

### Accuracy of AUCE-Based Method to Estimate EC_50_

We compared the results of the time-independent dose–response analysis to those obtained using a model-based approach (22). For this exercise, we considered the tumor cytotoxicity of two drugs with low (cibisatamab, *K*_D_ = 48.6 nM) and high (CEACAM5-TCB, *K*_D_ = 13.1 nM) binding affinities for the same tumor target (CEA). We tested the drugs on two different cell lines with low (CX1) and high (MKN45) target expression levels allowing for four separate comparisons.

**Fig. 3:**
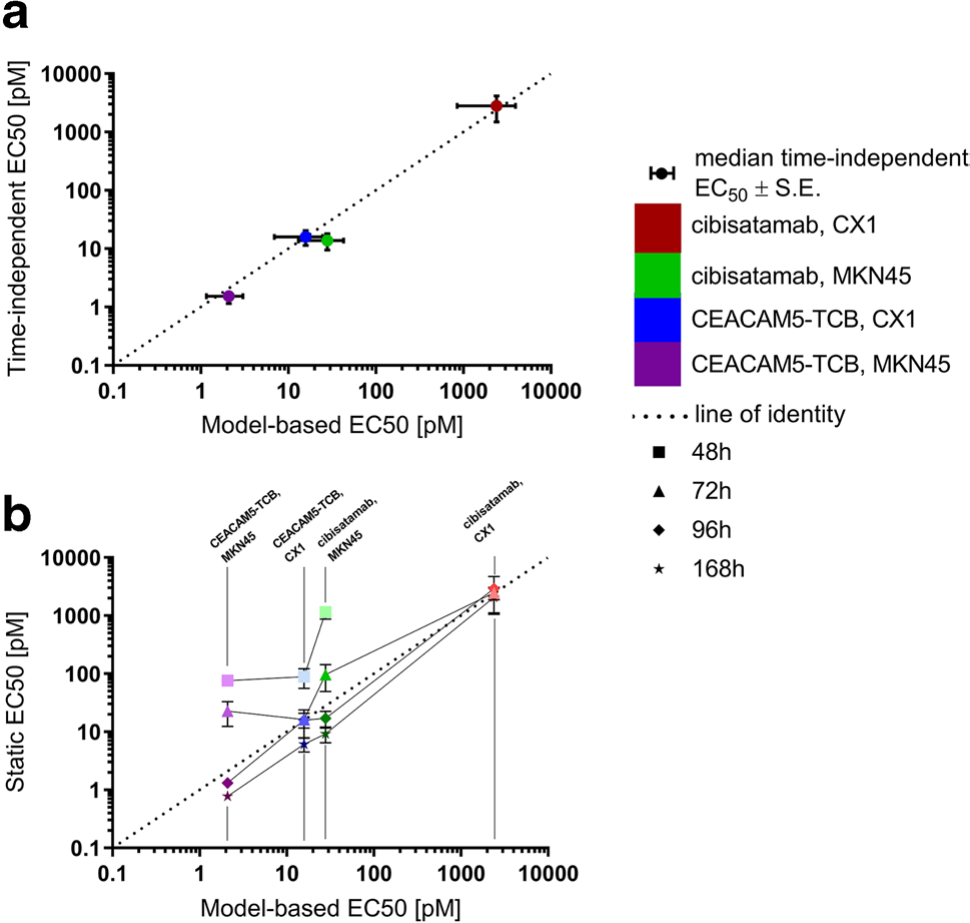
Comparison of tumor cell-killing potency (EC_50_) of cibisatamab and CEACAM-TCB, tested on CX1 and MKN45 tumor cell lines derived by modeling (displayed on the x-axis; %RSE are shown as horizontal error bars): **a** time-independent EC_50_ derived from AUCE (displayed on the y-axis; %RSE is shown as vertical error bars) or **b** static EC_50_ per given time point (displayed on the y-axis; % RSE is shown as vertical error bars)

We monitored the drugs’ effect on perturbation of tumor cell growth dynamics by measuring tumor cell count with FACS and used this as a basis to quantify tumor cell cytotoxicity and drug activity. We derived the model-based potency parameters by fitting the model (Eqs. (4)–(5)) to the observed tumor cell count data for all four scenarios. Figure 3a shows the EC_50_ estimates with the corresponding %RSE values represented as horizontal error bars. Since the mathematical model effectively captures the pharmacodynamic effects over time of all four scenarios and model-misspecification was excluded, we regard the estimated EC_50_ value as a good approximation of the “reference” potency, which can be used as a benchmark for the static and time-independent analysis. The estimated parameters and %RSE values are summarized in supplemental table S1. The model fit and observed data are shown in Figure S4.

Finally, we assessed the accuracy of the AUCE-based analysis and compared the corresponding EC_50_ values from all four tested scenarios to those estimated by model fitting (Fig. 3a). All four EC_50_ values are at the line of identity, suggesting good agreement between the AUCE-based potency values and the model-derived method. The same method was applied to assess the accuracy of the static approach considering all four time points (Fig. 3b). In three out of the four scenarios tested, the variability of the potency estimates between the time points spanned multiple orders of magnitude; 25% of values deviated more than fivefold from the model-based estimate.

### Time Course Analysis Using Transfected Cell Lines

**Fig. 4:**
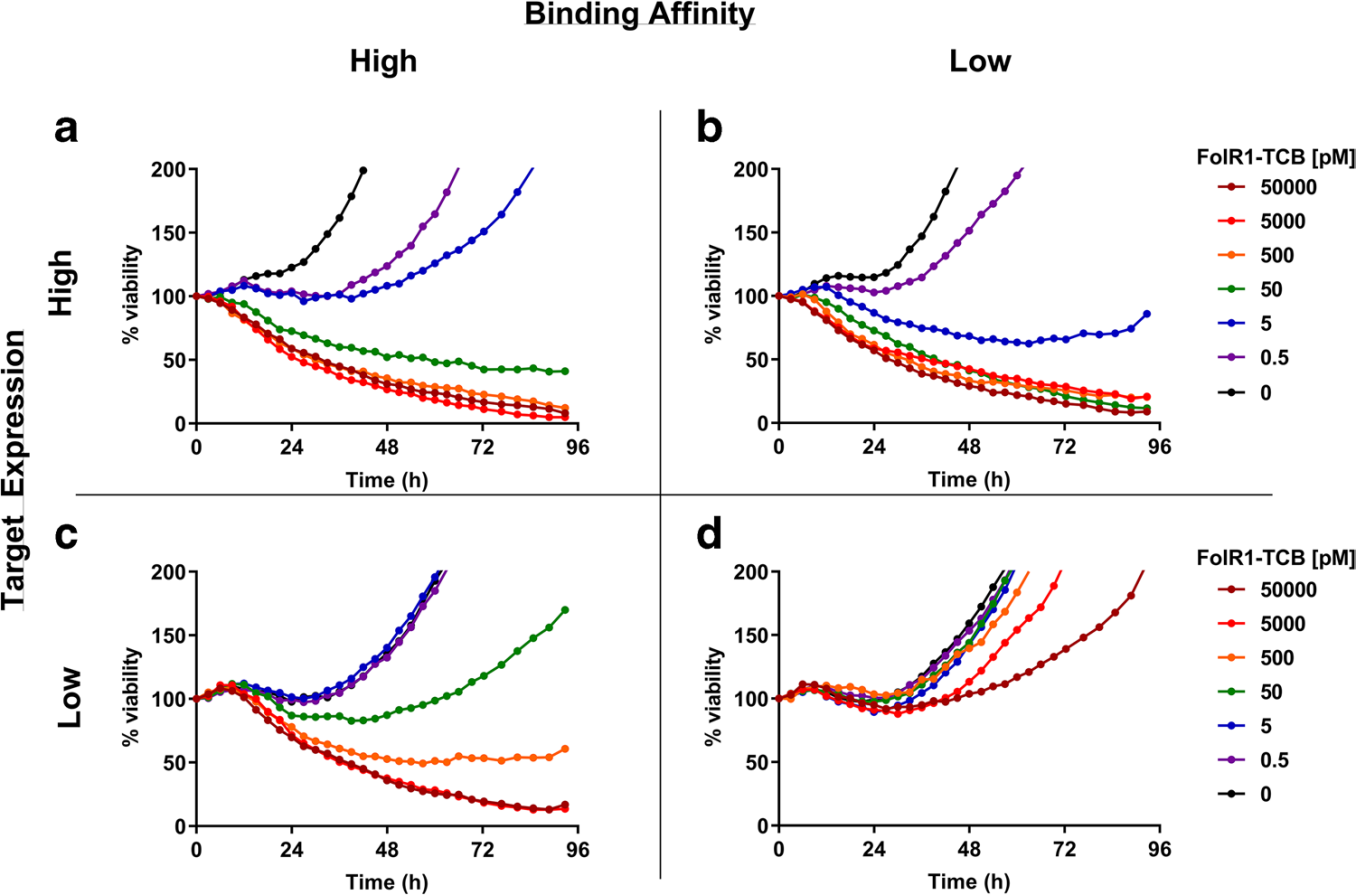
Time course profiles of tumor cells treated with high (**a**, **c**) and low (**b**, **d**) affinity FolR1-TCB on HEK transduced cell lines with high (**a**, **b**) and low (**c**, **d**) FolR1 expression. For better visualization of the higher doses, the y-axis is truncated at 200% viability. The complete plots can be found in Figure S6. The profiles of cytokine release are summarized in Figure S7

As a next step, we tested an *in vitro* image-based TDCC kinetic assay that also incorporated cytokine kinetic profiling as a less labor-intensive alternative to flow cytometry-based methods. We measured tumor cell cytotoxicity via live-cell imaging (incuCyte), which enables real-time visualization of viable tumor cells transfected with a red fluorescent protein (RFP) with fluorescence microscopy at standard cell culture conditions (humidified, 37 °C, 5% CO_2_). We tested two different CD3-bispecific drugs with high (*K*_D_ = 2.2 nM) and low (*K*_D_ = 60 nM) binding avidity for FolR1 (FolR1-TCBs) and assessed their pharmacological activity on RFP-transfected HEK cells with low (20,000 FolR1/cell) and high (505,000 FolR1/cell) expression levels co-cultured with PBMCs from a healthy donor. Figure 4 shows the time course profiles of tumor cell cytotoxicity reported as percent viable cells. In all tested scenarios, there was a clear dose–response. We observed maximum tumor cell cytotoxicity in all cases except when the low-affinity FolR1-TCB was incubated with the low FolR1-expressing cell line, which did not result in either measurable tumor cell cytotoxicity or cytokine release. In all four cases, we observed an early decrease in viability occurring over the first 24 h followed by a recovery and regrowth of the tumor cells except for the higher concentrations. The time course of different readouts as well as the time that it takes to reach the maximal effect (*T*_*max*_) varies across readouts and test systems (Fig. 1, Table II, Supplemental table S2). As a consequence, the estimated potencies may also vary across the different time points, as illustrated for TNFɑ (Supplemental figure S5).

**Fig. 5:**
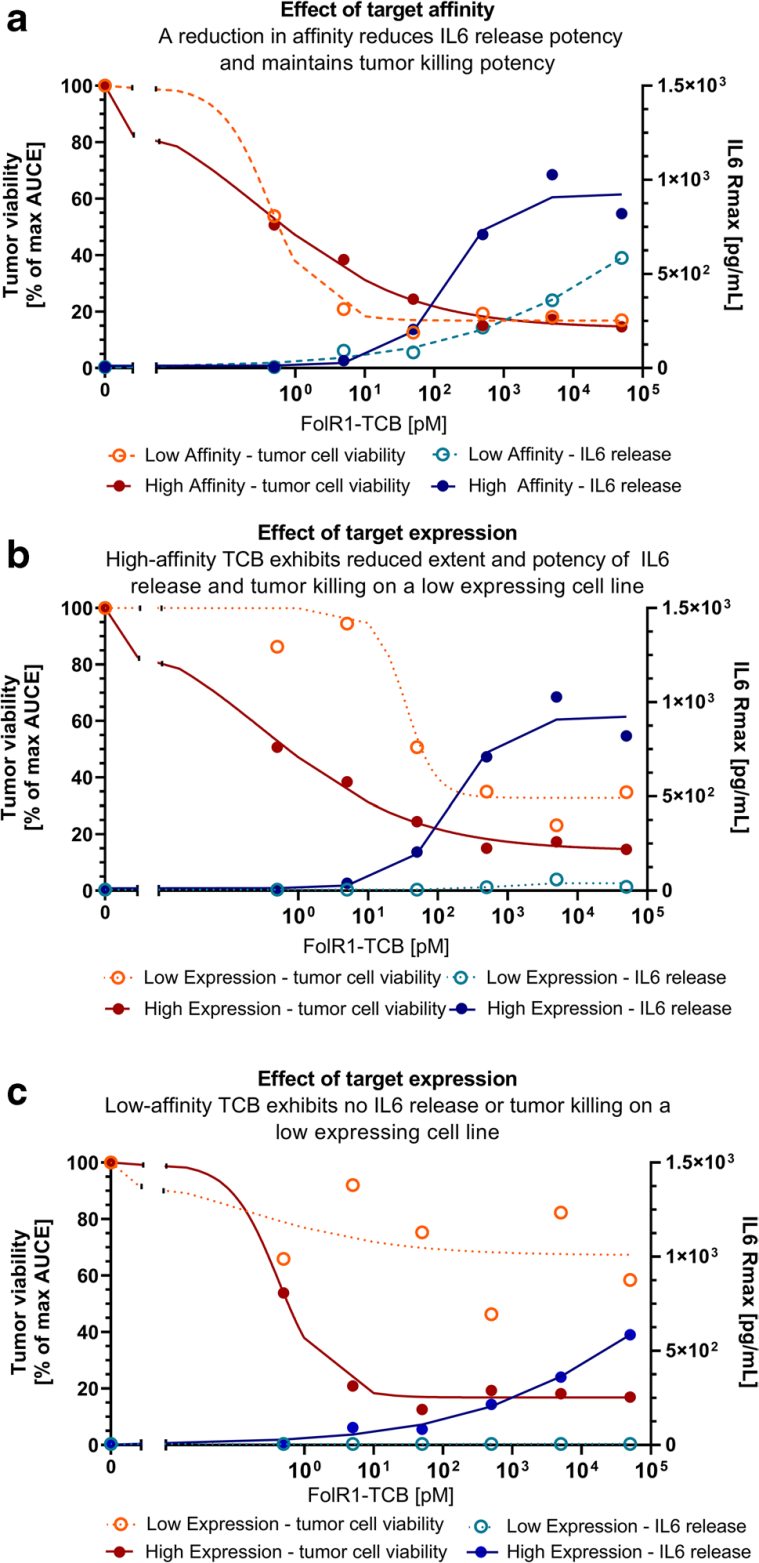
Time-independent dose-responses of high- and low-affinity FolR1-TCB variants tested in high- and low-expression cell lines. Dose–response of tumor cell cytotoxicity is expressed as the cumulative effect of tumor cell viability normalized to the control group (% of max AUCE, orange symbols, left y-axis) and dose–response of IL6 release is shown as the maximum response (*R*_*max*_, blue symbols, right y-axis). Symbols have been overlaid with the sigmoidal model fit of the data (solid/dotted lines). **a** Head-to-head comparison of high-affinity FolR1-TCB (solid circles) and low-affinity FolR1-TCB (open circles) tested on a high-expressing tumor cell line. **b** Head-to-head comparison of dose–response with high-affinity FolR1-TCB tested on a high (solid circles) and low (open circles) expressing cell line. **c** Head-to-head comparison of dose–response with low-affinity FolR1-TCB tested on a high (solid circles) and low (open circles) expressing cell line

The time span between the decrease in viability and the recovery was different for each scenario. The longest time to regrowth was observed for the high-affinity FolR1-TCB on a high expression cell line. In addition, we quantified the dose-responses of all four scenarios with the time-independent analysis of cytokine release. These results are summarized in Table III and displayed in Fig. 5.

**Table III:** AUCE Dose–Response Analysis of FolR1-TCB Affinity Variants Tested on Transfected HEK Cells with High and Low FolR1 Expression

Test system	Tumor cell cytotoxicity EC_50_ (pM) (%RSE)	Hill (%RSE)	IL6 release, EC_50_ (pM) (%RSE)	Hill (%RSE)
Low-affinity FolR1-TCB, low target expression	N.I	N.I	N.I	N.I
High-affinity FolR1-TCB, low target expression	30.1 (63)	1 (57)	N.I	N.I
Low-affinity FolR1-TCB, high target expression	0.42 (15)	1.3 (35)	1026 (36)	0.63 (18)
High-affinity FolR1-TCB, high target expression	0.28 (43)	0.35 (22)	116 (26)	1.3 (23)

*N.I.*, not identifiable; *na*, not applicable

As an illustrative example of how to assess a molecule’s therapeutic index, we compared the dose-responses of efficacy and safety readouts of the high- and low-affinity variant of FolR1-TCB on high and low target-expressing cells. Here, we again used tumor cell cytotoxicity as a readout for efficacy. For safety, we used IL6 release, assuming that, in this case, IL6 release is a relevant safety marker of on-target toxicity. When comparing the dose–response on the high expression cell line (Fig. 5a), both affinity variants have a similar potency for tumor lysis whereas the high-affinity compound has an eightfold higher potency for IL6 release associated with an approximately twofold higher maximum release (higher *E*_*max*_).

As a second illustrative example of how to assess the selectivity of a compound between high and low target expression, we compared the changes in pharmacological activity of both FolR1-TCB variants based on target expression (Fig. 5b and c). The high-affinity variant (Fig. 5b) induced maximal tumor lysis in both cell lines, whereas the low-affinity variant (Fig. 5c) showed selective tumor cell cytotoxicity with no effects on the low-expression cell line and maximal cytotoxicity in the high expression cell line.

More specifically, the high-affinity FolR1-TCB was approximately 100-fold more potent towards the high expression tumor cell line compared with the low-expression line (Fig. 5b). Furthermore, a 40-fold higher maximum IL6 response was observed for the high expression cell line. For the low-expression cell line, a sigmoidal model could not be fitted. A threshold concentration for IL6 release was observed to be 500 pM. The dose–response analysis of low-affinity FolR1-TCB targeting high- and low-expression cells (Fig. 5c) showed that there was only minimal tumor lysis and no IL6 release in the low-expression cell line, suggesting that low-expression tissues would be minimally targeted by the low-affinity variant.

In addition, we calculated the theoretical trimeric complexes at the corresponding EC_50_ (table S3) of both binders in the high expression cell line (Eq. S1-S7, supplemental section S3). As a result, a 20-fold higher trimeric complex concentration is estimated for the high-affinity binder.

### Retrospective MABEL Dose Prediction for Cibisatamab

In order to illustrate the value of the time-independent PK/PD analysis, we conducted a retrospective dose prediction based on the dataset and results for cibisatamab presented in this manuscript (Fig. 2, Table II). The retrospective MABEL dose was based on PA_30%_ of IL6 release in the high CEA-expression cell line (MKN45), which was the same cell line as previously used to derive the FIH starting dose (19). The PA_30%_ of IL6 release was calculated (Eq. (2)), with the estimated EC_50_ and the corresponding Hill coefficient (Table II). The MABEL dose is predicted to result in a *C*_*max*_ (maximal serum concentration) that corresponds to this pharmacological readout. Assuming that the dose of cibisatamab dissolves initially in the human serum with a typical volume of 3000 mL (29), a predicted MABEL dose of 450 μg is obtained (Table IV).

**Table IV:** Comparison of a Retrospective Dose Calculation Based on Time-Independent Experiments and the Actual First-in-Human Dose Applied in Clinics

Parameter	Retrospective dose prediction	Applied FIH dose
Definition of MABEL	*C*_*max*_ corresponding to PA30, IL6 release in MKN45*	*C*_*max*_ corresponding to EC20 of cytotox in MKN45 at 48 h^***^
EC20 (ng/mL)	100	46^***^
PA30 (ng/ml)	150	80^****^
Assumed human plasma Volume (mL)^**^	3000	3000
MABEL dose (μg)	450	50^***^

^*^Data presented in Fig. 1 and Table II; ^**^Davies et al*.* (29), ^***^reported FIH starting dose (Dudal et al. [19]), ^****^back-calculated from EC20, using Eq. (2) and assuming a Hill coefficient of 1

## DISCUSSION

In the present study, we propose a simple yet comprehensive method for accurately quantifying the pharmacological activity of CD3-bispecific antibodies. The cascade of events that lead to drug-induced cytotoxicity and cytokine release (11–13) can be investigated *in vitro* as a basis for determining the MABEL dose for FIH clinical trials (17). In a recent FDA guidance (30) on the development of bispecific antibodies with agonistic properties, reference is made to the FDA’s retrospective dose prediction of CD3-bispecifics (17) using *in vitro* assays. These are often done at a single time point, which can lead to a variation in the apparent potency of a given compound because of differences in incubation time and time point selection bias (23). In the present work, we found that this variation could span several orders of magnitude (Fig. 3b). We also showed that this bias could be circumvented by estimating the drug’s potency based on the dynamic effect over time, a time-independent metric, which is computed as the area-under-the-effect-curve. We demonstrated a reliable and accurate potency estimate using this method by cross-validating it with a model-based estimation.

In line with earlier observations (14–16), we found that the kinetics of the various pharmacodynamic processes triggered by CD3-bispecifics differ, such as tumor cell killing, T cell activation, and cytokine release. Based on these findings, we conclude that it is impossible to find a single optimal time point for all readouts. Instead, we suggest monitoring the various PD readouts over time and comparing these with their respective AUCE-based potency value in order to gain a more holistic understanding of the drug’s pharmacological activity.

**Fig. 6:**
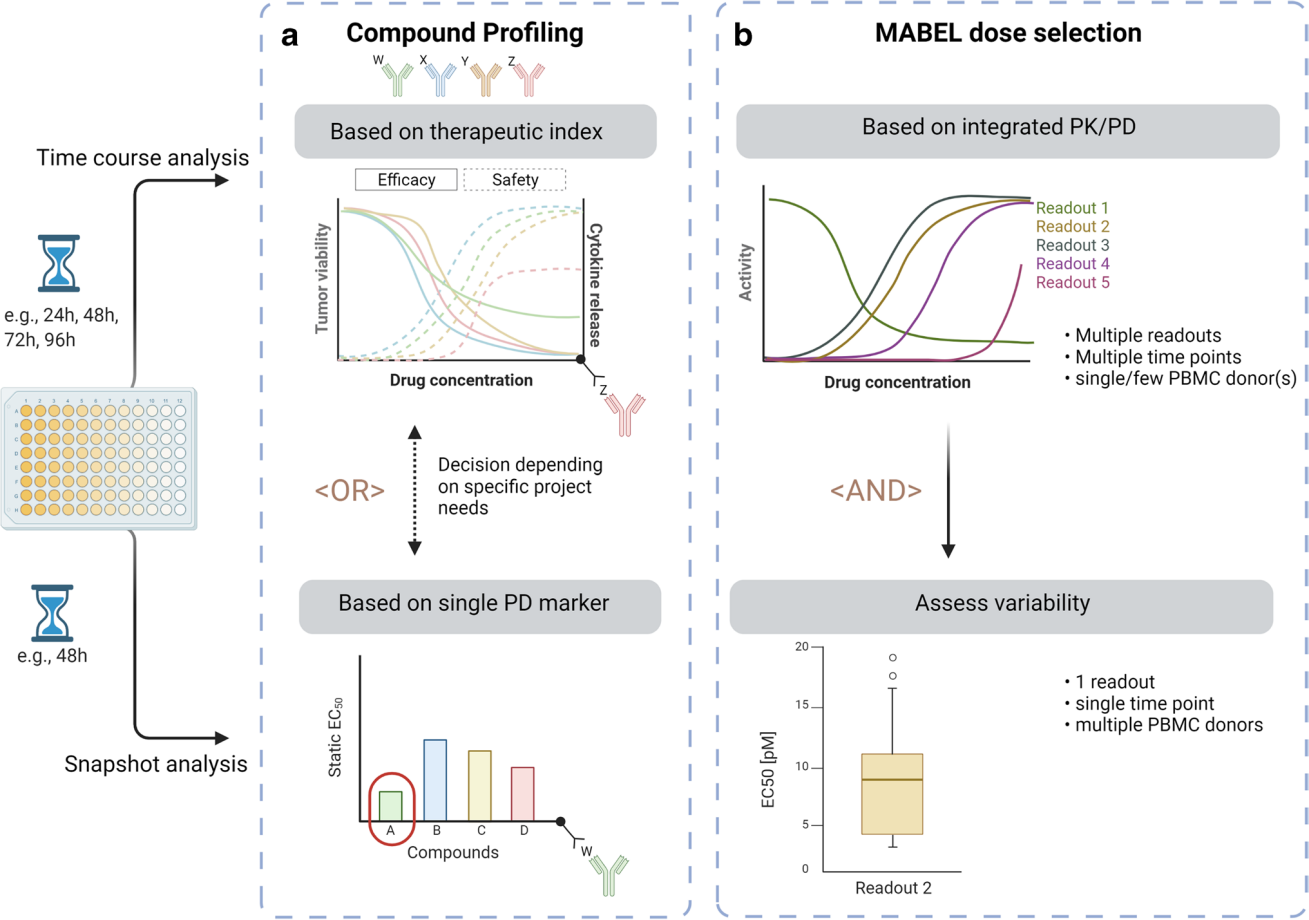
A schematic overview of strategic overview and decision tree for the complementary use of time-independent and static analyses in the early development of CD3-bispecifics is provided. **a** Two alternative options are depicted for compound profiling and candidate selection (*e.g.*, compounds W, X, Y, or Z). The upper panel illustrates compound selection based on the most favorable *in vitro* safety/efficacy balance applying a time-independent analysis on multiple readouts. The lower panel shows a static analysis based on a single readout. **b** For MABEL dose selection, a stepwise approach is proposed. First, a time-independent analysis is suggested to enable comparison across various readouts without the risk of time bias. This can be conducted with one or only a few PBMC donors (upper panel). For quantification of the donor-to-donor variability and to reduce the overall work package, the PD endpoint of interest can subsequently be tested with multiple PBMC donors at a single time point informed by the time-independent analysis (lower panel)

As observed in the presented datasets with cibisatamab, CEACAM5-TCB, and high- and low-affinity FolR1-TCBs, the time it takes for the PD readouts to reach their peak effect (*T*_*max*_) varies across different readouts and test systems (supplemental table S2) and is often not known a priori. We therefore propose a tailored approach to enable an integrated PK/PD analysis of readouts that occur on different timescales (Fig. 6) and show it can be applied in drug discovery and development. For drug candidate selection, there are two options proposed. This is either done based on a single PD readout (*e.g.*, potency on tumor cytotoxicity) and with a static analysis or based on the anticipated therapeutic index, in which case, time course analysis of the corresponding safety and efficacy readouts is recommended (Fig. 6a).

The exposure–response relationship of readouts, including potency, steepness of response, and maximal effect is expected to differ between test systems. Prerequisites for a thorough pharmacological assessment are the selection of the appropriate test systems as well as the appropriate design of the assay. This will enable the investigators to derive integrated quantitative insights and to select a relevant PD readout for the MABEL-based dose prediction. Together with information on the target biology in a healthy and diseased context as well as other supporting data, a time-independent *in vitro* analysis could provide a more rational basis to select and justify relevant readouts for a starting dose selection with minimal pharmacological activity and lower risk for adverse effects. This justification needs to be done on a case-by-case basis and will be supported by the integrated quantitative analysis. An important point to consider for the dose–response analysis is that not every concentration-dependent increase of effect can be captured with a sigmoidal *E*_*max*_ model. For those cases, we suggest estimating a threshold concentration at which minimal effects are expected (26). Especially in the context of adverse effects, this can be utilized to calculate the anticipated exposure margins (20) and to potentially give guidance on the dose-escalation scheme.

In order to demonstrate its utility in the context of FIH dose selection, we have conducted a retrospective MABEL prediction for cibisatamab and compared it to the clinical data (31). The actual MABEL starting dose was originally determined as the EC_20_ of tumor cytotoxicity with a static analysis (19). Here, we evaluated the exposure–response of efficacy (cytotoxicity) *versus* safety (IL6 release) with the integrated analysis in the same high-expressing cell line (MKN45) as previously utilized (19) to ensure the safety of patients with high tumor target expression.

The proposed analysis confirms that for cibisatamab, tumor cell cytotoxicity was the most sensitive readout as defined on the estimated potency value (EC_50_). Furthermore, it is suggested that cibisatamab has a favorable therapeutic index when comparing IL6 release to cytotoxicity in the MKN45 cell line (Fig. 2c). Based on this integrated *in vitro* PK/PD assessment, a PA_30%_ on IL6 release is selected as a basis for the MABEL dose of 450 μg. It was ninefold higher than the original starting dose of 50 μg and—when comparing it to clinical data—with an acceptable safety profile. This was 50-fold below the dose (2.5 mg) at which pharmacological activity was observed in clinics and ~ 80-fold below the reported MTD of 400 mg (31). In summary, the proposed MABEL approach is safe and may reduce the number of patients exposed at subtherapeutic dose levels during dose escalation.

In order to tailor and simplify this approach, we proposed a workflow where the first detailed time course analysis of various readouts is conducted with only one or a few PBMC donors in order to assess the potency and maximal response for each readout in a time-independent fashion (Fig. 6b). For quantification of the donor-to-donor variability and to reduce the overall work package, the PD endpoint of interest can subsequently be tested with multiple PBMC donors at a single time point. With such a stepwise approach, the process can be efficiently adapted and tailored based on the specific needs and questions for a given project.

This is applicable in early CD3-bispecific discovery and development in order to select favorable tumor-selective compounds tailored to the target of interest, to explore the therapeutic index of different molecules, or to define and assess the ideal compound properties for a given therapeutic target (4, 5, 32). Most tumor targets considered for therapeutic applications are overexpressed in tumor tissue and exhibit lower target expression levels in most healthy tissues, which allows for selective targeting of tumor cells with limited cytotoxicity in normal tissue (33). In these cases, the goal is to identify CD3-bispecifics with favorable compound properties that selectively kill tumor cells while exhibiting limited or no cytotoxicity to non-targeted tissue. To illustrate this, two compounds with different binding affinities for the same epitope on a both high- and low-expression target cell lines were compared with regards to their pharmacological profiles. Here, we highlight the utility of real-time imaging systems such as incuCyte or real-time cellular impedance like xCELLigence (34) that generate richer datasets and are less labor- and time-intensive than analogous workflows that use flow cytometry (35) in early drug discovery. The generation of time course data is especially important since cytotoxicity kinetics may differ between cell lines as we observe in the present study and has been reported for TCR-like CD3-bispecifics (36). In addition, the presented case example shows the value of a data-driven approach for compound selection. Here, a binder with higher affinity did not result in higher potency on tumor cytotoxicity as one may have anticipated based on in silico prediction that relate the formation of trimeric complexes to cytotoxicity (21, 37). The time-independent analysis of the high- and low-affinity FolR1-TCB revealed similar potency (EC_50_) values, but a steeper dose–response curve and a more favorable therapeutic index for the low-affinity binder. Further investigations are needed to better understand the various factors that trigger cytotoxicity beyond the formation of trimeric complexes (25).

Time-independent analysis enables the meaningful quantitative characterization of *in vitro* experiments, provided that the experimental design is appropriate. An informative dose range includes doses that span from minimum to full effect and a tailored observation period that captures the time course of the PD readouts of interest. However, these TDCC assays are dependent on the experimental conditions, such as the source of human PBMCs (*e.g.*, isolated PBMCs, whole blood, frozen/fresh PBMCs, purified T cells), the use of adherent or soluble cancer cell lines, the absolute number as well as the concentration of individual cell types, the PBMC donor-to-donor variability, and which can hamper robust quantification of the pharmacological activity. Another important consideration to *in vitro* experiments is the effector-to-target (E:T) cell ratio. The physiological effector-to-tumor (E:T) ratio in patients’ tumors is not always known, highly variable, and will depend on the site of action (*i.e.*, blood *versus* solid tumors). For illustration, the anticipated E:T ratio for solid tumors is reported with 1:150 (21, 38, 39). However, it has been discussed that—for *in vitro* assays—higher E:T ratios (*e.g.*, 2:1, 5:1, 10:1) are needed to compensate for the short assay duration of only a few days (34). While the time course PK/PD approach is certainly an improvement over the static assessment, it has limitations. It does not provide a potency estimate independent of all of these assay conditions and it does not allow project the outcome of other scenarios such as predicting tumor cell cytotoxicity as a function of target expression (25, 37). Instead, it is suggested to use a model-based approach (as proposed by Chen et al*.* (15), Betts et al*.* (21), Jiang et al*.* (37)) to get a potency estimate that can possibly predict the response with varying E:T ratios or to predict other untested scenarios. However, this would require time course data, and therefore, the proposed experimental design is suitable for complementary analysis.

The proposed method of time-independent analysis can be seen as complementary to PK/PD modeling in the early development of CD3-bispecifics. It provides a pragmatic means of comparing efficacy and safety data without the risk of time bias. Time-independent therapeutic indices may prove to be an important asset when comparing compounds. In order to facilitate this analysis, an automated workflow has been developed that generates graphic and textual outputs. This allows scientists to analyze, plot, and evaluate the data. The framework is intended to help scientists conduct a holistic analysis of their data instead of focusing on a single readout or experimental condition. The results of this automated analysis should be examined, and, if needed, further analysis can be conducted to address any remaining questions.

## CONCLUSIONS

Our time-independent PK/PD analysis enables robust quantification of the pharmacological activity of CD3-bispecifics and provides more accurate potency estimates than traditionally applied *in vitro* methods. We developed a leaner and less labor-intensive experimental protocol for the classical T-cell-mediated cytotoxicity assay for monitoring the time course of tumor cell cytotoxicity and cytokine release with real-time imaging. We also created a semi-automated workflow to quantify the pharmacological response. Improved *in vitro* assays and analysis methods may increase their translational relevance and pave the way for less animal experimentation. The proposed method enables head-to-head comparison of drug candidates based on their anticipated therapeutic index and may improve the identification of relevant FIH dose estimations of CD3-bispecifics.

## Supplementary Information

Below is the link to the electronic supplementary material.

Supplementary file1 (PDF 1.90 MB)

Supplementary file2 (XLSX 107 KB)

Supplementary file3 (XLSX 104 KB)

Supplementary file4 (PY 19 KB)

Supplementary file5 (PY 6 KB)
