# Supplementary material for: A Novel Approach for Quantifying the Pharmacological Activity of T-Cell Engagers Utilizing In Vitro Time Course Experiments and Streamlined Data Analysis
Source: AAPS J. 2021 Dec 3;24(1):7. doi: 10.1208/s12248-021-00637-2 (PMC8817205; doi:10.1208/s12248-021-00637-2)
Supplement: Supplementary file 1 — Supplementary file1 (PDF 1.90 MB) [file 12248_2021_637_MOESM1_ESM.pdf]

# A novel approach for quantifying the pharmacological activity of T-cell engagers utilizing *in vitro* time-course experiments and streamlined data analysis

Arthur Van De Vyver, Miro Eigenmann, Meric Ovacik, Christian Pohl, Sylvia Herter, Tina Weinzierl, Tanja Fauti, Christian Klein, Thorsten Lehr, Marina Bacac, Antje-Christine Walz

*Contents:*

Supplemental sections: 3
Supplemental tables: 3
Supplemental figures: 7

## Supplementary section S1

### S1.1 Materials, cell lines, and reagents

#### Materials

X-tremeGENE 9 DNA Transfection Reagent (ref. 6365779001), histopaque-1077 solution (ref. 10771) and hexadimethrine bromide (Polybrene, ref. H9268) were purchased from Sigma-Aldrich. Puromycin (ref. ant-pr-1) was purchased from InvivoGen. The APC-conjugated anti-FolR1 antibody (ref. LS-C129132) was purchased from LSBio. IncuCyte NucLight Rapid Red (ref. 4717) and IncuCyte CytoLight Rapid Green (ref. 4705) were purchased from Essen Bioscience. The cytokine detection kits for Cytokine Bead Array against IL2 (ref. 558270), IL6 (ref. 558276), IL10 (ref. 558274), IFNγ (ref. 558269), and TNFα (ref. 558273) were purchased from BD Biosciences. Millipore 0.22um Millex-GP filters (ref. SLGP033NS) were purchased from Merck. Plastic 48-well flat bottom plates (ref. 92148) were purchased from TPP (Trasadingen, Switzerland).

**Cell lines, expression vector and test items**

Human cancer cell lines CX1 (AAC129) and MKN45 (AAC409) were purchased from DSMZ, and HEK 293T cells (CRL-3216) were purchased from ATCC. Identity authentication was performed on MKN45 by Microsynth AG in 2018. No authentication was performed on CX1 or HEK 293T cells. Mycoplasma testing was performed on HEK 293T cells. Human peripheral blood mononuclear cells (PBMCs) were isolated from fresh blood from healthy donors via a histopaque-1077 density gradient.

ViraSafeTM lentiviral packaging system (Cell biolabs, cat# VPK-206) was used for viral production. The plasmids were pRSV-Rev packaging vector (Part No. 320022), pCgpV packaging vector (Part No. 320024), and pCMV-VSV-G envelope vector (Part No. 320022).

**S1.2 Lentivirus production and transduction of target cell line**

Full-length cDNA encoding FolR1 was cloned into a lentiviral transfer vector and used to transfect HEK 293T cells along with packaging constructs pRSV-Rev, pCgpV, and pCMV-VSV-G by using the X-tremeGENE 9 DNA Transfection Reagent according to the manufacturer's protocol. The virus-like particles were harvested 3 days after transfection. The supernatant was centrifuged to remove any cellular debris. Assembled lentiviral particles were isolated by filtration with a 0.22 µm Millex-GP filter and stored at -80°C.

Transduction of HEK 293T cells was performed in 48-well flat bottom plates that were pre-seeded with $1x10^3$ viable cells the day before. The culture medium was replaced with 320µL purified supernatant containing the virus-like particles together with 80µL fresh culture medium (DMEM + 10% FCS) and 4 µg/mL hexadimethrine bromide.

The cells were spinoculated for 2h (800g, 32°C) in aerosol tight centrifugation buckets in order to increase transduction efficiency. The cells were exposed to the lentiviral vectors for another 16h at standard culture conditions (37°C, 5% $CO_2$), followed by washing and addition of fresh medium. Two days after transduction, 1 µg/mL Puromycin was added. After initial selection, the cells were sorted for low (designated $FolR1^{low}$) and high (designated $FolR1^{high}$) surface expression of FolR1 by using the BD FACS ARIAIII Cell Sorter (BD Bioscience). The sorted cells were cultured to establish stable clones and to guarantee monoclonality. The FolR1 expression density was confirmed and tested for stability by performing flow cytometric analysis with an APC-conjugated anti-FolR1 antibody over a period of 4 weeks.

## Supplemental Section S2

### Automated dose-response analysis with Python

A semi-automated workflow has been developed in Python in order to perform time-independent analyses. The AUCE calculations, curve fitting, simulations, and data plotting were conducted using several libraries including *scikit-learn*, *scipy*, *numpy* and *matplotlib*. The *curve fit* function uses nonlinear least squares optimization. Outputs are the optimal values for the parameters, so that the sum of the squared residuals of model output and data as well as the variance-covariance matrix are minimal. The parameter estimates were provided along with their RSE% values. These can be used to flag any identifiability issues with the model parameters. Not all the profiles will fit appropriately with the models explored in this analysis. Nevertheless, the automated analysis provides all the necessary information to investigate whether the model fits and the parameters are reliable. The outputs include plots of raw data per experimental conditions (denoted by the readout and experimental condition; e.g., 'CD8_CD25 over time cibisatamab CX1.jpeg'), $EC_{50}$

estimations at different time points (denoted ‘Potency Change Over Time’), $EC_{50}$ estimation across different experimental conditions including different cells lines and different drugs (denoted ‘Cumulative Potency’), sigmoidal model fits for each experimental condition (denoted ‘Model Fit’), estimated sigmoidal model parameters for the model fits including RSE% values (Sigmoidal Model Parameters.txt), simulations for sigmoidal fits for each experimental condition (Sigmoidal Model Simulations.xls), calculated AUCE values (AUCE.xls) as well as Hockey-Stick model fits (denoted with *HOCKEYSTICK*), estimated model parameters including RSE% values (Hockey Stick Parameters.txt), and simulations for Hockey stick fits for each experimental condition (Hockey Stick Model Simulations.xls).

## Supplemental Section S3

### Calculating quasi-equilibrium trimer concentration

The quasi-equilibrium equations for calculating the trimer concentration in function of drug concentration, binding affinities and target availability have been derived by Schropp and colleagues. In short, the following equations are required to calculate the trimer concentration at quasi-equilibrium (Eq. S1-S7). $KD_1$ is the binding affinity between the drug ($C$) and the free tumor target ($R$). $KD_2$ is the binding affinity between $C$ and free CD3 ($CD3$). $R^0_{tot}$ and $CD3^0_{tot}$ are the total tumor target and CD3 concentration at time 0, respectively. Receptor concentrations are calculated by multiplying the expression density with the total concentration of receptor expressing cells. These concentrations are converted into nmol/L through dividing by the Avogadro constant ($N_a$; $6.022*10^{23}$ molecules/mole):

$$Concentration_{Receptors} = \frac{Density_{Receptors} * Concentration_{cells}}{N_a} * 10^9$$

Quasi-Equilibrium calculations (Eq. S1-S7):

$$aa = \left(1 + \frac{C}{KD_1}\right) * \frac{C}{KD_1*KD_2} \quad \text{(Eq. S1)}$$

$$bb = C * \frac{(R_0^{tot} - CD3_0^{tot})}{KD_1*KD_2} + \left(1 + \frac{C}{KD_1}\right) * \left(1 + \frac{C}{KD_2}\right) \quad \text{(Eq. S2)}$$

$$dd = -CD3_0^{tot} * \left(1 + \frac{C}{KD_1}\right) \quad \text{(Eq. S3)}$$

Equations S1-S3 are required to calculate the concentration of free R and CD3 (Eq. S4-S6):

$$R = \frac{R_{tot}^{0}}{1 + \frac{C}{KD_1} + \frac{CD3*C}{KD_1*KD_2}} \quad \text{(Eq. S4)}$$

$$CD3 = \frac{(-bb + \sqrt{(bb^2 - 4*aa*dd)})}{2*aa} \quad \text{(for C > 0)} \quad \text{(Eq. S5)}$$

$$CD3 = CD3_{tot}^{0} \quad \text{(for C = 0)} \quad \text{(Eq. S6)}$$

The trimer concentration can then be calculated based on the concentration of free drug (C), free R and free CD3, and the respective binding affinities. The explicit equation for trimer concentration at quasi-equilibrium becomes:

$$Trimer = \frac{C*R*CD3}{KD_1*KD_2} \quad \text{(Eq. S7)}$$

The quasi-equilibrium model above assumes rapid binding and a constant receptor pool ($R_{tot}(t) = R_{tot}(0)$, $CD3_{tot}(t) = CD3_{tot}(0)$). A Python script is provided in the GitHub repository (https://github.com/PKPD-coder/time-independent_analysis_in_vitro.git) to calculate the

steady-state trimer concentration at the specified drug concentrations. The trimer concentration can be converted into an average number of trimers per tumor cell (Eq. S8).

$$Trimer_{Tumor} = \frac{(Trimer*10^{-9})*N_a}{Tumor} \qquad \text{(Eq. S8)}$$

With *Trimer* the total trimer concentration [nmol/L], $N_a$ the avogadro constant, and *Tumor* the total concentration of tumor cells in the assay [cells/L].

**Table S1: Model-derived EC50 parameter estimates and Coefficient of variation (%CV)**

| **Scenarios** (Affinity - Expression level) | **EC50 (%RSE) [pM]** | **kmax (%RSE)** | **tau (%RSE) [h]** | **kg (%RSE) [1/h]** | **K (%RSE) [cells/uL]** |
|---|---|---|---|---|---|
| Cibi CX1 (low-low) | 2400 (64.7) | 0.0895 (13.2) | 15 (13.2) | 0.128 (3.9) | 295 (6.1) |
| Cibi MKN45 (low –high) | 28.0 (53.4) | 0.133 (26.6) | 22.8 (20.5) | 0.0658 (6.6) | 367 (10.1) |
| CEACAM5 CX1 (high-low) | 15.8 (56.3) | 0.148 (7.6) | 12.5 (11.2) | 0.145 (5.38) | 258 (15.1) |
| CEACAM5 MKN45 (high-high) | 2.1 (44.6) | 0.114 (16.5) | 18.5 (16.5) | 0.0686 (7.17) | 414 (10.8) |

**Table S2: summary of time points at which maximal effect was observed ($T_{max}$) for each experiment**

| Test system | cibisatamab | | CEACAM5-TCB | | FolR1-TCB High affinity | | FolR1-TCB Low affinity | |
|---|---|---|---|---|---|---|---|---|
| | MKN45 | CX1 | CX1 | MKN45 | High expression | Low Expression | High expression | Low Expression |
| **Assay** | 96-well plate @ 200µL<br>30’000 target cells<br>300’000 PBMCs | | | | 96-well plate @ 200µL<br>10’000 target cells<br>100’000 PBMCs | | | |
| **Time for maximal effect (h)** | | | | | | | | |
| IL2 | 24 | nd* | 24 | 24 | 94 | nd* | 68 | nd* |
| IL6 | 72 | nd* | 96 | 72-96 | 94 | 68 | 94 | nd* |
| IL10 | 48 | nd* | 48 | 48 | 94 | 94 | 94 | 94 |
| TNF$\alpha$ | 24 | nd* | 24 | 24 | 94 | nd* | 94 | nd* |
| IFN$\gamma$ | 48 | nd* | 48-72 | 48-72 | 94 | nd* | 94 | nd* |
| Activated cytotoxic T-cells ($CD25^+$ $CD8^+$) | 96 | 168 | 96 | 96 | | | | |
| $CD4^+CD25^+$ | 96 | 96 | 96 | 96 | | | | |
| $CD4^+PD1^+$ | 168 | 72 | 168 | 168 | | | | |
| CD4+TIM3+ | 168 | 24 | 168 | 168 | | | | |
| Tumor cyto-toxicity | 96 | 168 | 168 | 96 | 94 | 94 | 94 | 24 |

$T_{max}$, time of maximal response; nd, not determined

* no cytokine release was detected

**Table S3. Theoretical trimer concentration at half-maximal tumor killing in various test systems**

| Test system | Theoretical concentration of trimers formed at $EC_{50}$ of cytotoxicity [pM] | Theoretical average number of trimers per tumor cell at $EC_{50}$[a] |
|---|---|---|
| High affinity FolR1-TCB, low target expression | $5e^{-4}$ | 6 |
| Low affinity FolR1-TCB, high target expression | $6.6e^{-6}$ | 0.08 |
| High affinity FolR1-TCB, high target expression | $1.2e^{-4}$ | 1.4 |
| cibisatamab, MKN45 | $1.24e^{-3}$ | 5 |

Theoretical trimer concentration calculated from the $EC_{50}$ of tumor cytotoxicity reported for cibisatamab (table 2) and FolR1-TCB variants (table 3). [a] the theoretical concentration of trimers converted into an average number of trimers per tumor cell in the co-culture (see Eq. S8)

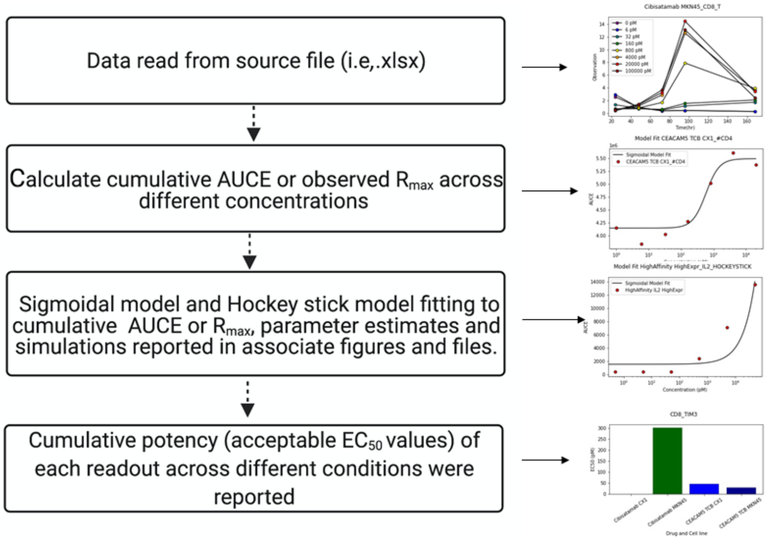


**Figure S1.** Flowchart of the automated workflow developed for dynamic PK/PD analysis in Python. The program reads a user-provided data sheet then plots each readout or condition over time for all reported drug concentrations. The cumulative AUCE is computed for each readout and for all reported drug concentrations. The AUCE dose-responses are then used to fit a sigmoidal model. A hockey-stick model is simultaneously fitted to determine a threshold concentration in order to attempt to provide a usable pharmacology metric in case a sigmoidal model cannot be fitted. Finally, the dynamic potency or threshold concentrations are reported in *.txt* format for each readout and experimental condition.

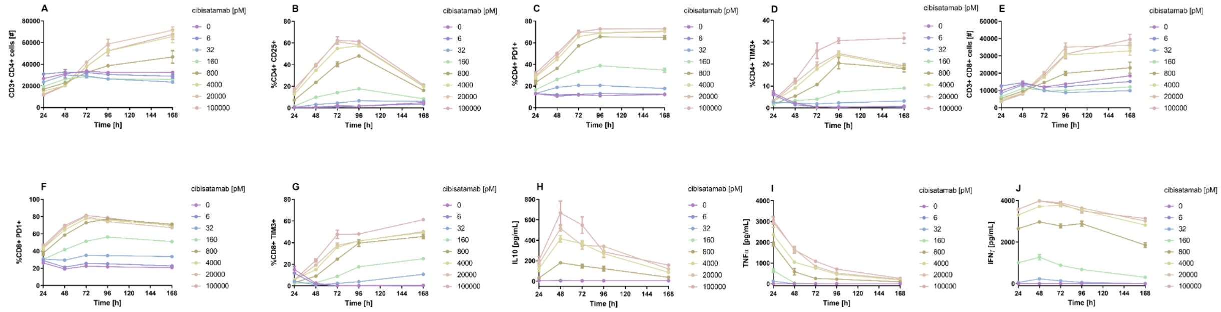


**Figure S2.** Dose-response profiles over time of CD4 T-cells (A) and the positive fractions of CD25+ (B), PD1+ (C), TIM3+ (D), and CD8 T-cells (E) and the positive fractions of PD1+ (F) and TIM3+ (G), and cytokines IL10 (H), TNFα (I), and IFNγ (J), at 8 different cibisatamab

concentrations targeting the tumor cell line MKN45. Readouts presented as mean + SEM. Cytokine concentrations below the lower limit of quantification (LLOQ) of the CBA kits were set as 1/2 LLOQ (5 pg/mL).

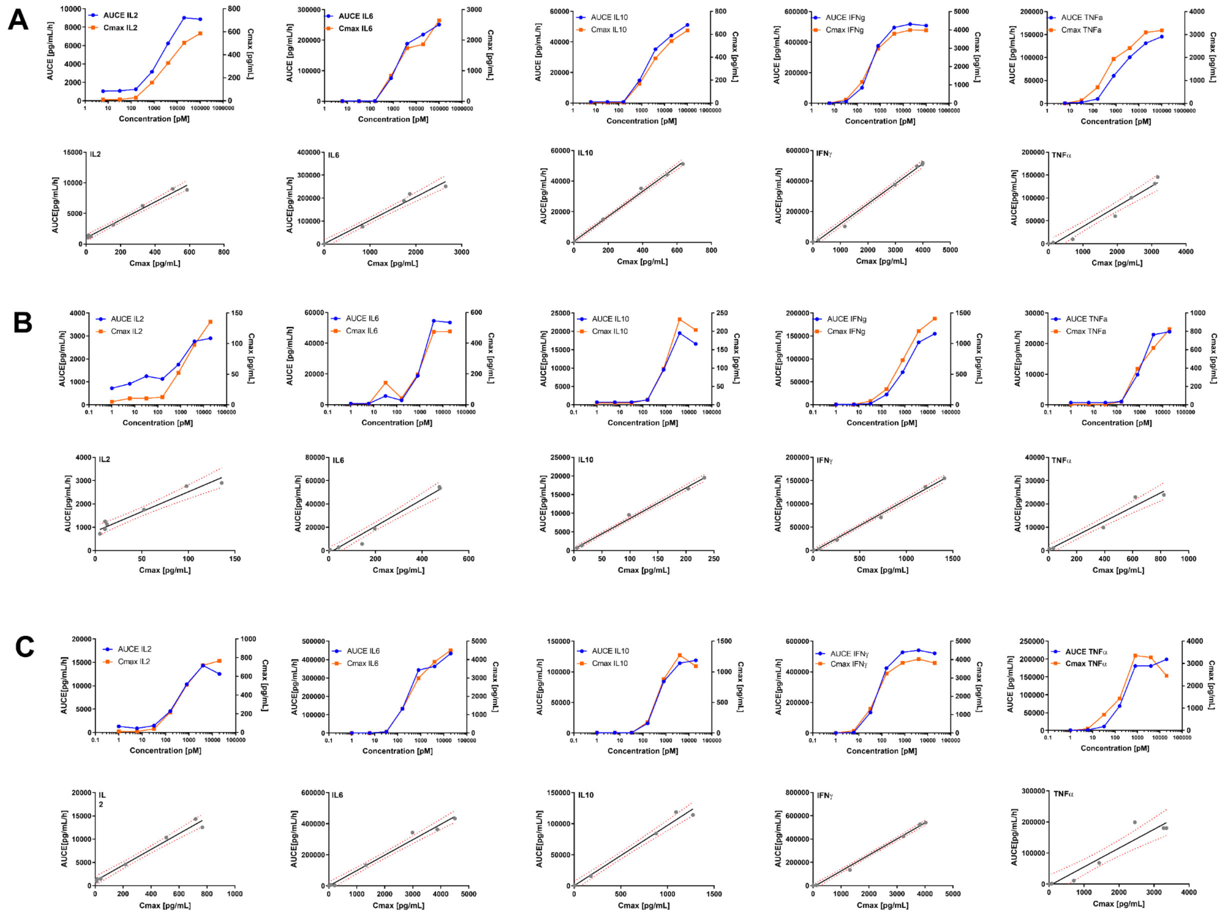

**Figure S3.** Comparison of computed AUCE (blue) versus Cmax (orange) for the release of (from left to right) IL2, IL6, IL10, IFNγ, and TNFα for A. cibisatamab on MKN45 cells; B. CEACAM5-TCB on CX1 cells; and C. CEACAM5-TCB on MKN45 cells in function of TCB concentration (top figures). Linear regression between AUCE and Cmax (bottom figures). The solid black line represents the best fit. Red dashed lines correspond to the 95% confidence interval.

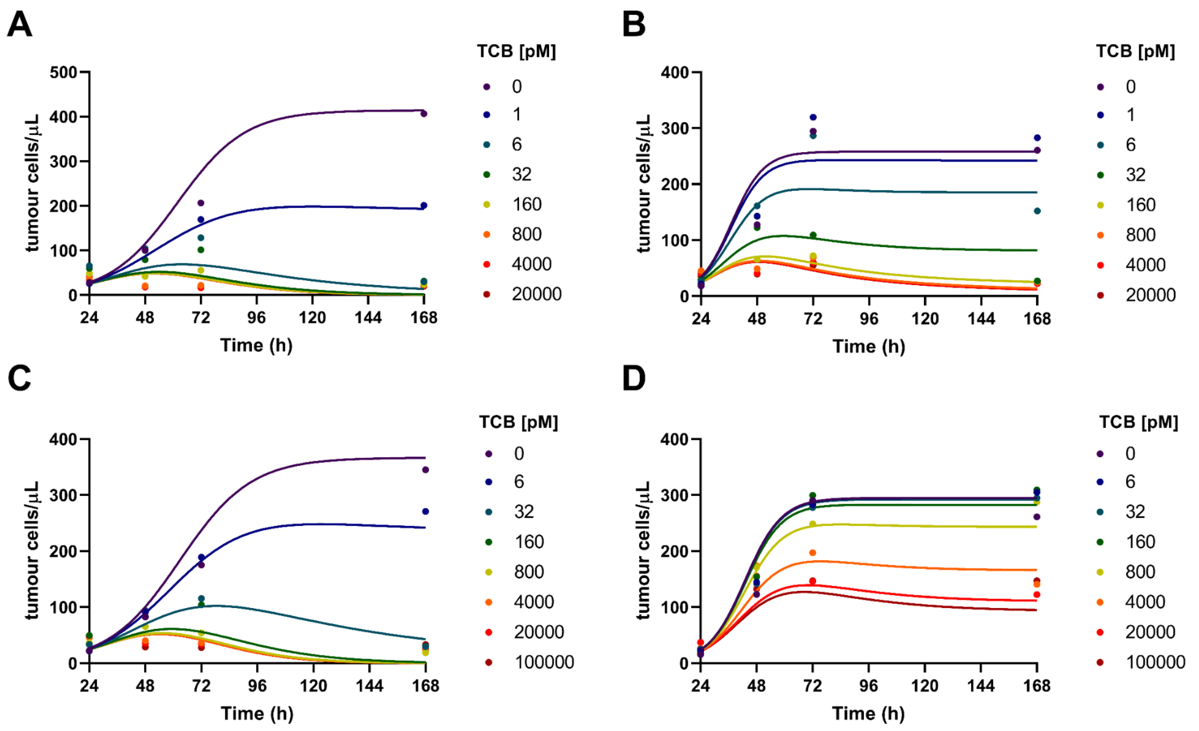


**Figure S4.** Model fits (lines) of tumour cell counts over time (dots) of A. CEACAM5-TCB targeting MKN45; B. CEACAM5-TCB targeting CX1; C. cibisatamab targeting MKN45; D. cibisatamab targeting CX1.

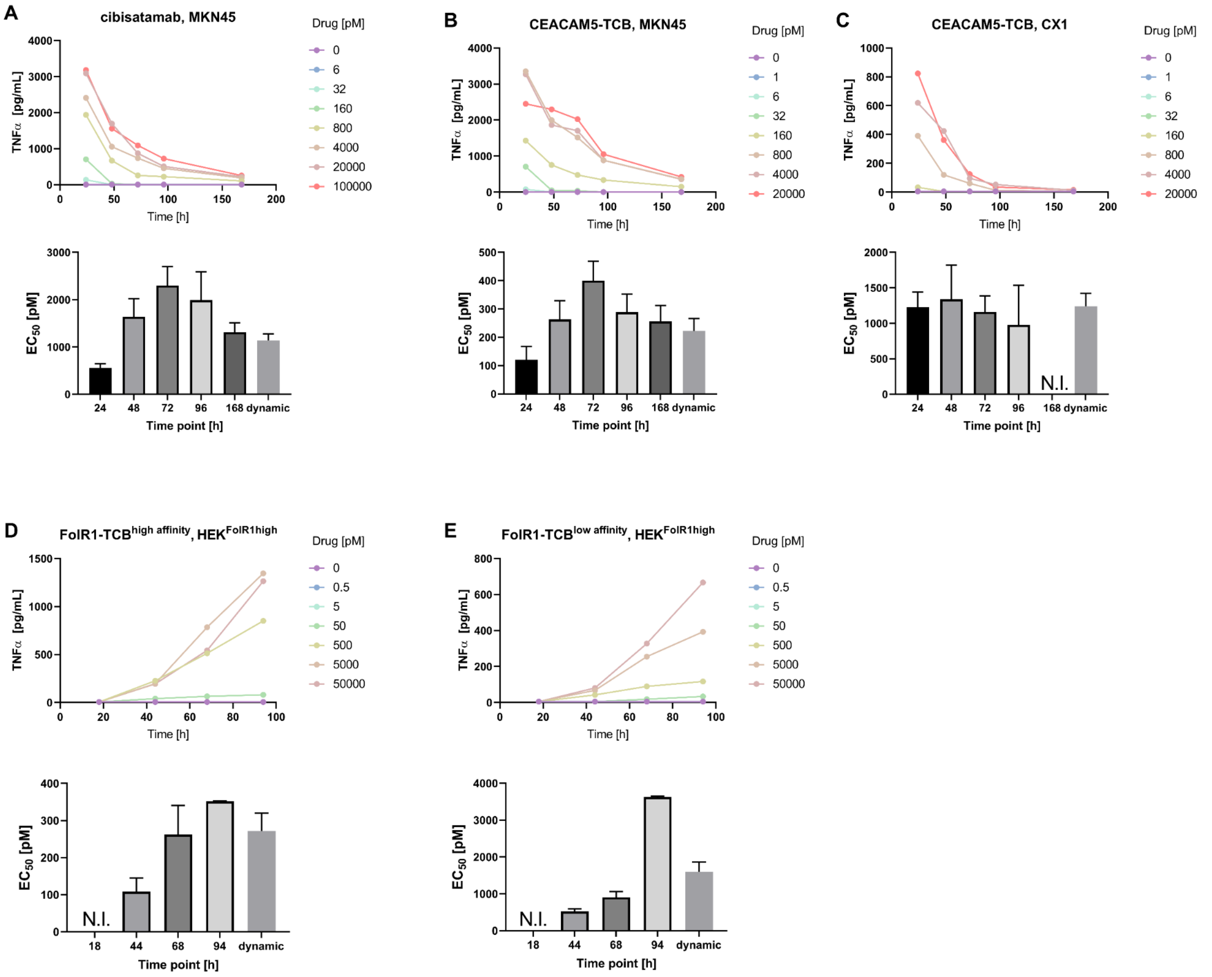


**Figure S5.** Differences in time course between different test systems; here exemplified by TNFα release, shown as TNFα concentration over time (top) and potency changes over time (bottom). Both cibisatamab (**A**) and CEACAM5-TCB (**B-C**) treatment elicit an early peak in TNFα release, followed by a rapid decline in concentration. Conversely, FolR1-TCB high (**D**) and low (**E**) affinity variants targeting a high expressing cell line elicit a build-up of TNFα over time, reaching an apparent maximum at the last measured time point. No TNFα release was observed for cibisatamab targeting CX1 or FolR1-TCB variants targeting the low expressing cell line (not shown).

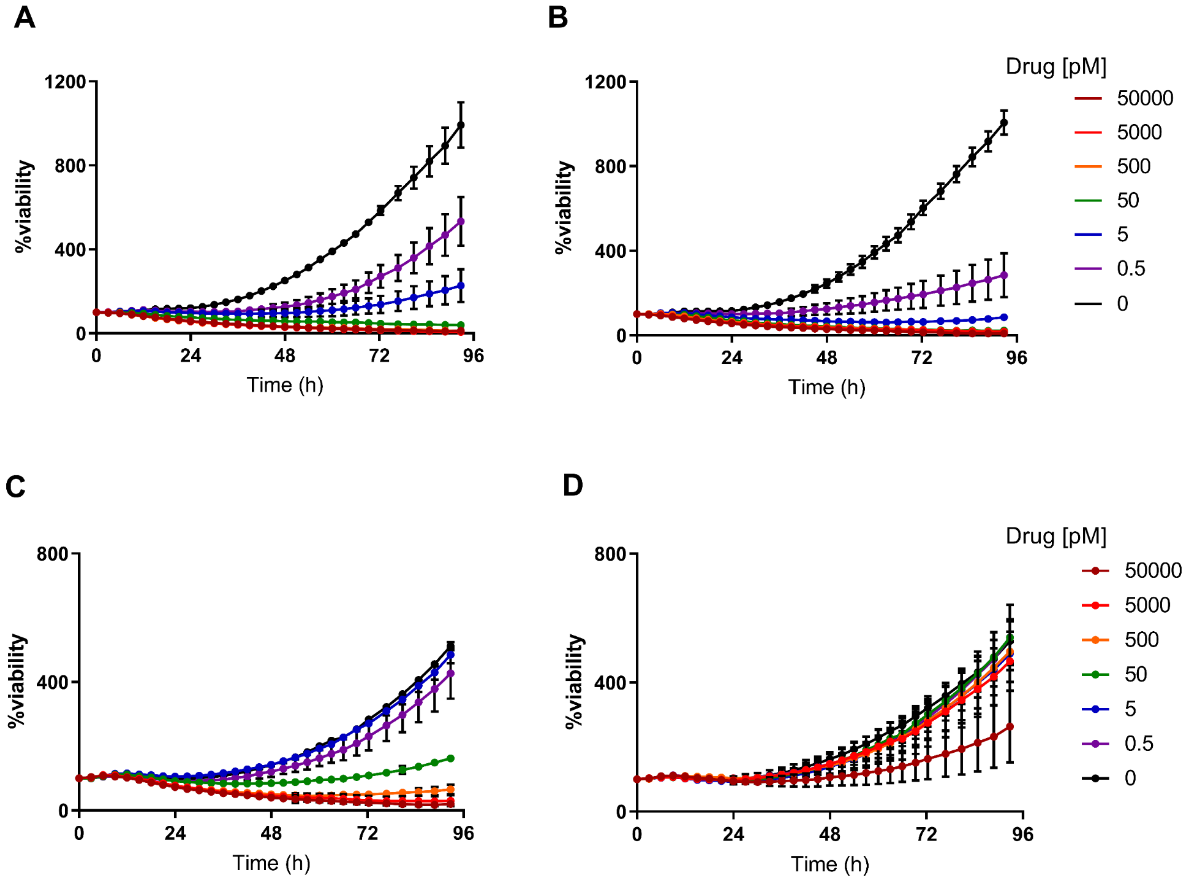


**Figure S6.** Tumor time-course profiles of FolR1-TCBs reported as mean ± SEM. High-affinity FolR1-TCB targeting **A.** FolR1$^{high}$ cell line and **C.** FolR1$^{low}$ cell line; low-affinity FolR1-TCB targeting **B.** FolR1$^{high}$ cell line and **D.** FolR1$^{low}$ cell line.

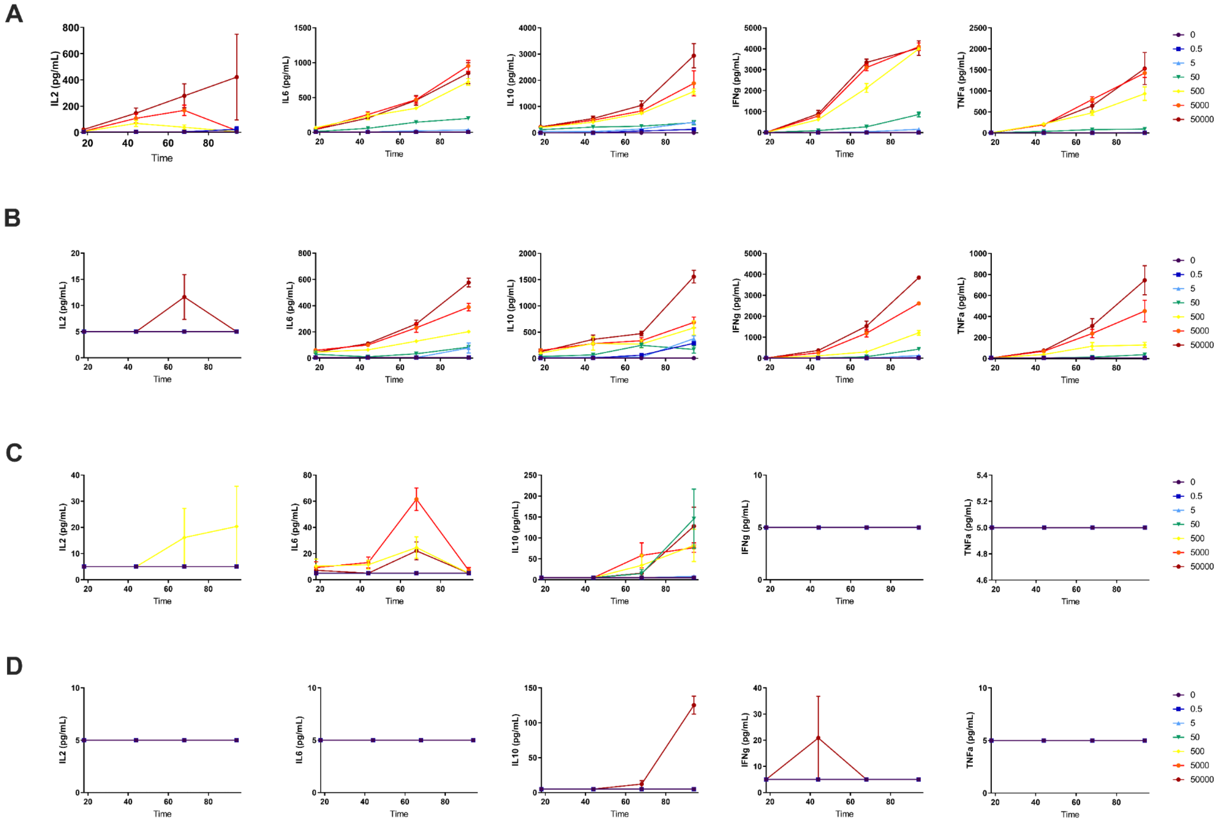


**Figure S7**. Cytokine time-course profiles of (from left to right) IL2, IL6, IL10, IFNγ, and TNFα from the in vitro tumor killing assays of high-affinity FolR1-TCB targeting A. FolR1high cell line and C. FolR1low cell line; low-affinity FolR1-TCB targeting B. FolR1high cell line and D. FolR1low cell line. Data reported as mean ± SEM.
